# Supplementary material for: Evaluation of Complementary Feeding Indicators Among Children Aged 6–23 Months According to the Health Literacy Status of Their Mothers
Source: Nutrients. 2024 Oct 18;16(20):3537. doi: 10.3390/nu16203537 (PMC11510195; doi:10.3390/nu16203537)
Supplement: Supplementary file 1 [file nutrients-16-03537-s001.zip › nutrients-3242233-supplementary.pdf]

Supplementary File S1. Sociodemographic characteristics of mothers and infants and the foods that the children ate the previous day

1. How old are you?
   ------
2. What is the type of your family?
   a) Nuclear family
   b) Extended family
   c) Other----
3. What is your educational status?
   a) Primary school
   b) Elementary school
   c) High school
   d) University
   e) Master’s degree/doctorate
4. What is your working status?
   a) Housewife
   b) Working
5. Where is your hometown?
   ----
6. How many children do you have?
   ----
7. How many people are in the household?
   ----
8. How is your family income?
   a) Income is less than the outcome
   b) Income equals to outcome
   c) Income is higher than the outcome

   Please answer the questions below for your older child if you have more than 1 child between 6-23 months.

9. How old is your baby? (Please write as months)
   ----
10. What was his/her gestational birth week?
    a)<38
    b)≥38
11. What is your baby’s gender?
    a) Girl
    b) Boy
12. Does your baby have an illness that she/he should take regular medication and follow up?
    a) No (please go to the 15th question)
    b) Yes
13. Please write about your baby’s illness.
    ----
14. Is your baby breastfed?

a) yes, she/he is still breastfed
b) she/he has been breastfed for a while but now she/he isn't
c) never breastfed

15. Is your baby using a pacifier?
    a) Yes she/he is
    b) She/he used but now not using
    c) Never used
16. Is your baby having bottle?
    a) Yes she/he is
    b) She/he used but now not using
    c) Never used
17. Is your baby fed formula?
    a) Yes she/he is
    b) She/he had for a while but now she/he isn't
    c) Never used
18. How many times did your baby fed formula in the last 24 hours (yesterday)?
    a) 0
    b) 1
    c) 2
    d) 3
    e) 4
    f) 5 and more
19. When did you give your baby a complementary feeding (any solid, semi-solid or soft food except breastmilk and formula) first?
    a) When she/he was 3 months or younger
    b) At 4th months
    c) At 5th months
    d) At 6th months
    e) At 7th months
    f) At 8th months
    g) Not started yet
20. How many times did your baby have complementary feeding in the last 24 hours (yesterday)?

a) 0
b) 1
c) 2
d) 3
e) 4
f) 5
g) 6 and more

21. Please tick off the foods and beverages that your child consumed in the last 24 hours.

- breast milk
- grains, roots, tubers and plantains
- pulses (beans, peas, lentils), nuts and seeds

- dairy products (milk, infant formula, yogurt, cheese)
- flesh foods (meat, fish, poultry, organ meats)
- eggs
- vitamin-A rich fruits and vegetables (like green or red vegetables, carrots, broccoli, yellow and red fruits)
- other fruits and vegetables.

22. Please tick off the foods and beverages that your child consumed in the last 24 hours.
    - Fruit juice, lemonade (homemade or sugar added)
    - Coke, soda pop, iced tea
    - Candies, chocolate, Turkish delight, and other sugar confections.
    - Frozen treats like ice cream, gelato, sherbet, sorbet, popsicles, or similar confections.
    - Cakes, pastries, sweet biscuits, and other baked or fried confections
    - Chips, crisps, cheese puffs,
    - None

Supplementary File S2. TSOY-32 Health Literacy Scale

The following items relate to various health-related issues. Please indicate how easy or difficult it is for you to perform each task.

#	Question	Very easy	Easy	Difficult	Very difficult	No opinion
1	When you have a health complaint, researching whether it is a symptom of a disease					
2	When you have a health complaint, reading and understanding any related literature (brochure, booklet, poster, etc.)					
3	When you have a health complaint, evaluating whether the advice from your family or friends is reliable					
4	When you want to go to a healthcare facility, researching which doctor to consult					
5	When you want to go to a healthcare facility, researching how to make an appointment (like booking an appointment)					
6	Making an appointment by phone or online when you need to visit a healthcare facility					
7	Researching and finding information about treatments for diseases that concern you					
8	Understanding your doctor's explanations about your illness					
9	Evaluating the advantages and disadvantages of different treatment options suggested by your doctor					
10	Taking your medications as prescribed by healthcare professionals (doctors, pharmacists, etc.)					
11	Understanding the instructions for using the medication on the package					

12	Deciding if you need to seek a second opinion from another doctor					
13	Understanding the information about preparation before tests/examinations (like following a diet)					
14	Researching and finding the location of the department (laboratory, clinic, etc.) you need in the hospital					
15	Deciding what to do in an emergency situation (like an accident or sudden health issue)					
16	Calling an ambulance when necessary					
17	Following your doctor's advice to get regular health check-ups					
18	Researching and finding information about conditions that can harm your health (like being overweight or having high blood pressure)					
19	Understanding health warnings related to conditions harmful to your health (like being overweight or having high blood pressure)					
20	Researching and finding information on how to cope with unhealthy behaviors (like smoking or insufficient physical activity)					
21	Understanding health warnings related to unhealthy behaviors (like smoking or insufficient physical activity)					
22	Researching and finding information about health screenings related to your age, gender, and health status (like breast cancer screenings for women, prostate cancer screenings for men)					

23	Understanding information recommended in sources like the internet, newspapers, TV, or radio for better health					
24	Deciding whether the information recommended in sources like the internet, newspapers, TV, or radio is reliable for better health					
25	Understanding information on food packaging that you think may affect your health					
26	Evaluating the positive and negative features of your living environment (home, street, neighborhood, etc.) that affect your health					
27	Researching what can be done to make your living environment (home, street, neighborhood, etc.) healthier					
28	Evaluating which of your daily behaviors (like exercising, eating healthy, not smoking) affect your health					
29	Changing your lifestyle (like exercising, eating healthy, not smoking) for your health					
30	Following a diet plan given in writing by a dietitian					
31	Giving advice to your family or friends on how to be healthier					
32	Interpreting changes in health policies					
